# Role of the mechanotransductor PIEZO1 in megakaryocyte differentiation

**DOI:** 10.1111/jcmm.70055

**Published:** 2024-09-20

**Authors:** Julien Demagny, Sonia Poirault‐Chassac, Damtz Nehemie Ilsaint, Aurore Marchelli, Cathy Gomila, Hakim Ouled‐Haddou, Louison Collet, Maïlys Le Guyader, Pascale Gaussem, Loïc Garçon, Christilla Bachelot‐Loza

**Affiliations:** ^1^ HEMATIM UE4666, University Picardie Jules Verne Amiens France; ^2^ Biological Hematology Department CHU Amiens‐Picardie Amiens France; ^3^ Université de Paris Cité, Innovative Therapies in Hemostasis, INSERM Paris France; ^4^ Service d'hématologie biologique Hôpital Européen Georges Pompidou, Assistance Publique‐Hôpitaux de Paris Paris France

**Keywords:** megakaryocytes, PIEZO1, proplatelet formation

## Abstract

From haematopoietic stem cells to megakaryocytes (Mks), cells undergo various mechanical forces that affect Mk differentiation, maturation and proplatelet formation. The mechanotransductor PIEZO1 appears to be a natural candidate for sensing these mechanical forces and regulating megakaryopoiesis and thrombopoiesis. Gain‐of‐function mutations of PIEZO1 cause hereditary xerocytosis, a haemolytic anaemia associated with thrombotic events. If some functions of PIEZO1 have been reported in platelets, few data exist on PIEZO1 role in megakaryopoiesis. To address this subject, we used an in vitro model of Mk differentiation from CD34^+^ cells and studied step‐by‐step the effects of PIEZO1 activation by the chemical activator YODA1 during Mk differentiation and maturation. We report that PIEZO1 activation by 4 μM YODA1 at early stages of culture induced cytosolic calcium ion influx and reduced cell maturation. Indeed, CD41^+^CD42^+^ numbers were reduced by around 1.5‐fold, with no effects on proliferation. At later stages of Mk differentiation, PIEZO1 activation promoted endomitosis and proplatelet formation that was reversed by PIEZO1 gene invalidation with a shRNA‐PIEZO1. Same observations on endomitosis were reproduced in HEL cells induced into Mks by PMA and treated with YODA1. We provide for the first time results suggesting a dual role of PIEZO1 mechanotransductor during megakaryopoiesis.

## 1 INTRODUCTION

PIEZO1 was first described in 2010 as a mechanotransductor in neuron‐derived cell line, leading to a strong interest in mechanotransduction for the scientific community.^1^ PIEZO1 is a large, three‐blade propeller‐shaped transmembrane protein encoded by the *FAM38A* gene that can be activated by various stimuli such as fluid shear stress, osmotic pressure, matrix stiffness or cell density.^2^, ^3^ They induce opening of the channel, resulting in a rapid cationic current upon activation. In this process, PIEZO1 senses and transforms mechanical forces into biochemical signals. Although non‐selective, PIEZO1 presents a preferential conductance for calcium ions (Ca^2+^).^4^ It is broadly expressed and its activation has been involved during development and in various physiological processes and cell systems such as vascular architecture, bone or red blood cells.

PIEZO1 gain‐of‐function mutations are known to cause hereditary xerocytosis (HX), a haemolytic anaemia due to red blood cell dehydration consecutive to the increase in intracellular Ca^2+^ which in turn activates the Gardos channel inducing potassium leak and water loss. Among its various roles, PIEZO1 also plays a role during early and late erythropoiesis. Indeed, we previously showed that PIEZO1 activation resulted in delayed erythroid differentiation in UT7‐EPO and primary cells depending notably on STAT5 and ERK1/2 phosphorylation status.^5^ Moura et al. showed that PIEZO1 activation due to GOF mutations in reticulocytes from HX patients delayed in vitro reticulocyte maturation, characterized by a slower loss of CD71 and RNA content.^6^ The function of PIEZO1 in haematopoiesis is not limited to erythropoiesis. Concerning the megakaryocytic lineage, PIEZO1 presence was evidenced by proteomic and transcriptomic analyses in platelets, primary megakaryocytes (Mks) and megakaryocytic cell lines.^7^, ^8^, ^9^ Moreover, fluid shear stress induced a Ca^2+^ influx in platelets that was inhibited by GsMTx4, an inhibitor of cationic mechanosensitive channels including PIEZO1. GsMTx4 reduced collagen‐induced thrombus formation under flow.^3^ This result was confirmed in vivo in mice treated daily with GsMTx4. In this model, GsMTx4 reduced platelet activation and thrombosis induced by hypertension.^10^

Environmental stiffness are important for megakaryopoiesis (Mkpoiesis), including ploidy and proplatelet formation.^3^, ^11^ However, a minimum of stiffness is important and necessary for megakaryocytic development as evidenced by 3D cultures that produce Mks with higher ploidy, higher development of demarcation membranes and more proplatelet formation than liquid culture.^11^

Various sensory actors have been identified in Mks, such as integrins or mechanosensitive receptors. Mechanosensitive receptors are known to play a role during Mkpoiesis, such as some members of the TRP channel family.^12^ Indeed, in K562 and HEL cells exposed to PMA, TRPA1 induced CD41 and CD61 expression and polyploidization.^13^ In a low stiffness matrix, TRPV4 activation enhanced platelet formation through integrin β1 activation and Akt phosphorylation.^14^ Mkpoiesis could thus be regulated through interactions between Mks and their direct environment via mechanoreceptors. Despite these converging arguments on the role of mechanotransdution in MKpoiesis, very few data have been published so far on PIEZO1 involvement in this process. In the megakaryocytic cell line Meg‐01, shear stresses induce PIEZO1‐dependent Ca^2+^ flux.^3^ Recently, it has been shown in human CD34^+^ cells and mice Mks that PIEZO1 activation decreased megakaryocytic maturation, altering both ploidy and plateletogenesis.^9^ Our study was designed to assess the role of chemical activation of PIEZO1 during early and late megakaryocytic differentiation. We used two cellular models, HEL cell line and primary CD34^+^ cells driven in vitro towards Mk differentiation. Using YODA1 and a shRNA based knock‐down (KD) strategy, we evaluated step by step the effects of PIEZO1 during Mkpoiesis, that is, the Mk commitment, the endomitosis process and proplatelet formation.

## 2 METHODS

Flow cytometry analysis and cell sorting, calcium flux assessment, lenti/retroviral production and cell infection, quantitative reverse transcriptase‐polymerase chain reaction, PIEZO1 western blot analysis and statistical analysis are detailed in Appendix S1.

### 2.1 Cell culture

CD34^+^ cells (HSC) from human cord blood and leukapheresis were sorted by an immunomagnetic bead cell‐sorting system (AutoMacs; Miltenyi Biotec) as previously described.^5^ Cells were cultured in serum‐free IMDM medium (Gibco‐Invitrogen) supplemented with BIT 9500 (StemCell Technologies), 1‐thioglycerol and liposomes (complete medium) for 7 days in the presence of stem cell factor (SCF, 20 ng/mL; miltenyi Biotec) and 50 nM TPO peptide agonist AF13948 (Sigma‐Aldrich). From Day 7 to Day 12, cells were further cultured with complete medium added with 20 nM TPO. HEL cell line was cultured in RPMI 1640 medium (Gibco‐Invitrogen) supplemented with 10% fetal bovine serum, 1% penicillin–Streptomycin (Sigma Aldrich) and 10 nM PMA. YODA1 (Sigma Aldrich) was pre‐diluted in DMSO before addition to the culture medium. Control and YODA1 conditions were at the same final DMSO concentration (0.01%) and renewed at each passing of the cells. Cell suspension was counted with Kova Slides or TC‐20 Automated cell counter (Bio‐Rad). Cell mortality was investigated with blue trypan TC‐20 Automated cell counter (Bio‐Rad) (cell suspension 1:2 diluted with blue trypan), DAPI staining solution (Miltenyi Biotec) by flow cytometry or with Colorimetric Cell Viability Kit IV (MTT) (Promokine).

### 2.2 Measurement of ploidy

At Day 7, CD41^+^CD42^+^ were sorted and plated in 96‐well plates at a concentration of 3.10^5^ cells/mL in the presence of YODA1 or control (DMSO). At Day 11 or 12, cells were resuspended in phosphate‐buffered saline (PBS) containing 0.1% BSA at 1.10^6^ cells/mL and fixed with 3 volumes of a cold solution of 70% ethanol. After incubation for 1 h at 4°C, cells were washed twice and resuspended in a DAPI solution (0.1 μg/mL). The mean ploidy was calculated by the following formula: (2 N × number of cells at 2 N ploidy) + (4 N × number of cells at 4 N ploidy) + (…) + (32 N × number of cells at 32 N ploidy)/total number of cells.^15^ Same protocol was used for the HEL. sh PIEZO1/scramble‐infected cells were fixed with the Cytofix/Cytoperm (BD Biosciences, San Jose, CA) kit to preserve GFP‐signal.

### 2.3 Quantification of Mks bearing proplatelets

CD41^+^CD42^+^ Mks were sorted at Day 7 and plated in 96 well‐plates at a concentration of 3000 cells/well in complete medium and 20 nM TPO. From Day 10 to Day 12, Mks bearing proplatelets were counted by inverted light microscopy at 20× objective. Within each experiment, at least 600 cells were observed in consecutive microscopic fields. The presence of at least one pseudopodia extension was considered as Mks bearing proplatelets. sh PIEZO1/scramble‐infected CD41^+^CD42^+^ Mks were sorted GFP^+^ at Day 9 and plated in 96 well‐plates. Mks bearing proplatelets were counted at Day 11 as above on at least 400 cells.

## 3 RESULTS

### 3.1 PIEZO1 is expressed and functional in human primary Mks

**FIGURE 1:**
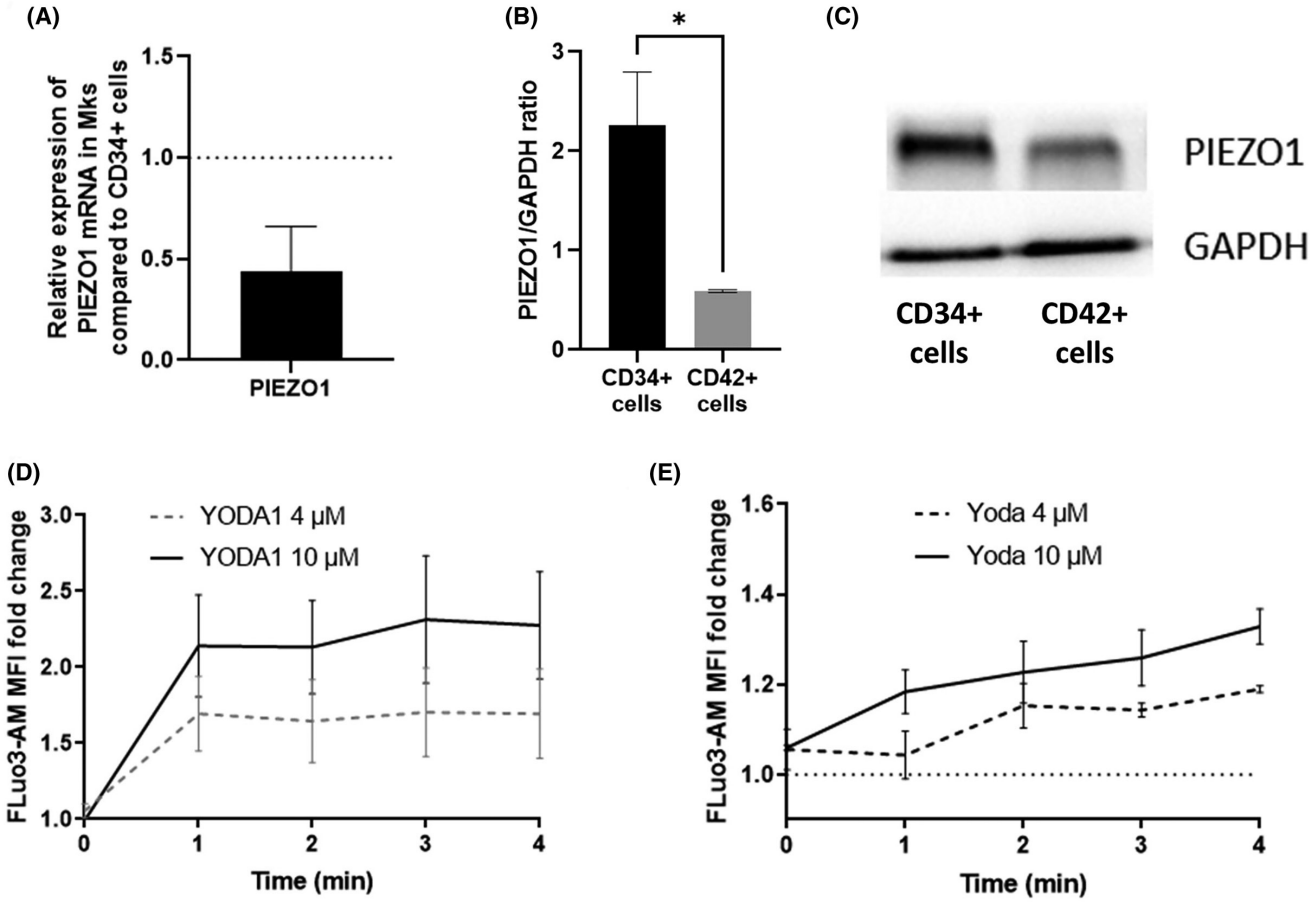
PIEZO1 expression in human primary CD34+ and Mks and Calcium flux activity. (A) Relative expression of PIEZO1 mRNA in CD41^+^ mature Mks at Day 12 of culture normalized on HPRT in Mks relative to CD34^+^ cells (*n* = 3). (B) Expression of PIEZO1 protein in CD34^+^ cells and in CD42^+^ mature Mks normalized on GAPDH in Mks (*n* = 4). (C) Representative western‐blot showing PIEZO1 and GAPDH protein expression in CD34^+^ cells and CD42^+^ mature. (D, E) Time course of cytosolic Ca^2+^ in response to four (dotted line) or 10 μM (solid line) YODA1 at Day 3 (D) or Day 12 (E) of megakaryocytic culture differentiation. Cells were labelled using Ca^2+^ probe Fluo3‐AM and mean fluorescence intensity (MFI) was recorded and expressed as fold change (normalized on DMSO) (*n* = 3 from 4 cord blood samples). *p < 0.05.

We assessed PIEZO1 expression at RNA and protein level during in vitro Mk differentiation from cord blood CD34^+^ cells. A shown Figure 1A–C, PIEZO1 levels decreased during Mk commitment and differentiation (CD42^+^ Mks vs. CD34^+^ cells). We then checked the presence and functionality of PIEZO1 on human cord blood CD34^+^ cells using YODA1, a specific chemical activator of PIEZO1.^16^ At Day 3 of culture, addition of 4 or 10 μM YODA1 induced an increase of cytosolic Ca^2+^ which reached a plateau at 1 min with an estimated 1.7 and 2.3 fold increase at 4 min, respectively (Figure 1D), compared to 5.6 fold increase in the presence of 5 μM ionophore (data not shown). This Ca^2+^ influx was milder at Day 12, that is, 1.2 and 1.3 fold (Figure 1E), in culture containing more than >80% CD41^+^ Mks (data not shown). Furthermore, the lack of Ca^2+^ increase in the presence of EGTA pre‐treatment is consistent with a Ca^2+^ influx induced by YODA1 and mediated by PIEZO1 (Figure S1).

### 3.2 PIEZO1 activation inhibits megakaryocyte differentiation of human cord blood CD34 ^+^ cells

**FIGURE 2:**
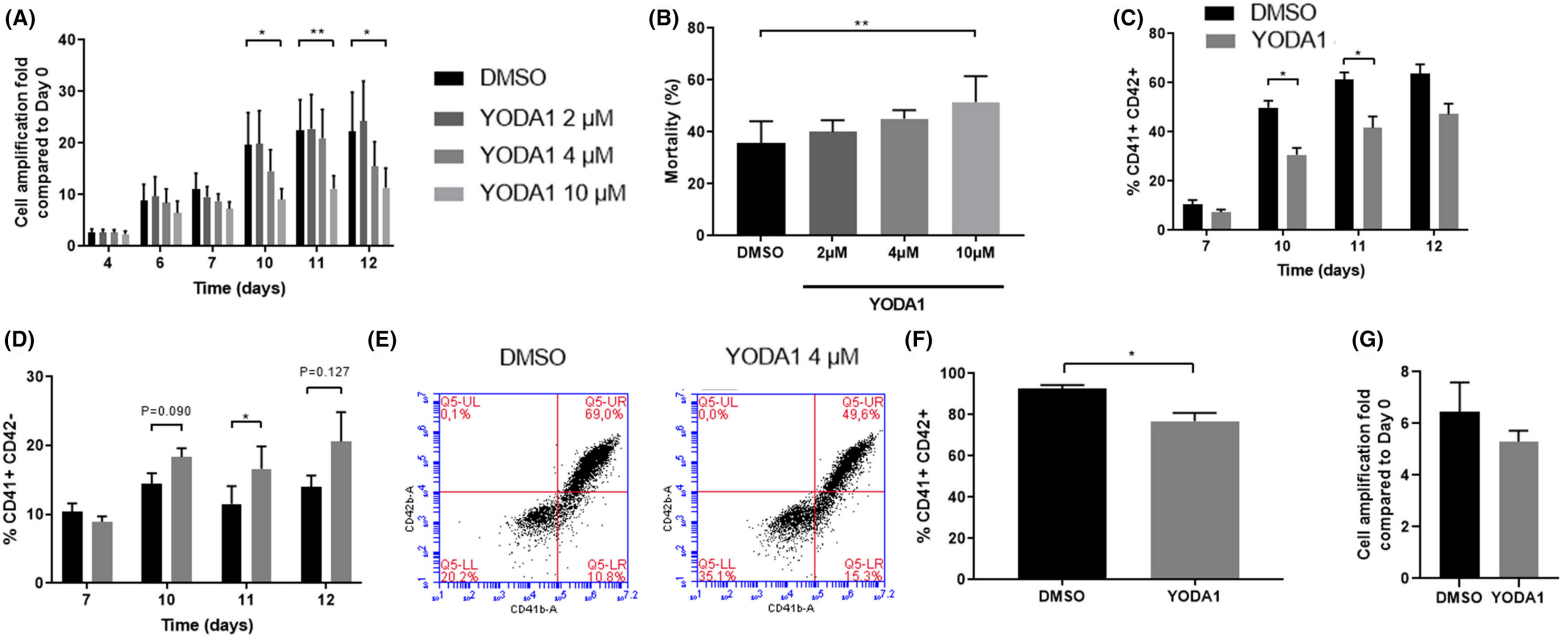
Effect of PIEZO1 activation on cell proliferation and megakaryocyte differentiation in human cord blood CD34^+^ cells. (A–E) Cell cultures were exposed from Day 0 to Day 12 to DMSO (control) or to 2 μM, 4 μM or 10 μM of YODA1 and were evaluated for: (A) cell proliferation at Days 4, 6, 7, 10, 11 and 12. Results are expressed as cell amplification fold compared to Day 0 (*n* = 6 from 12 cord blood samples); (B) mortality rate assessed by trypan blue staining at Day 12 (*n* = 6 from *n* = 12 cord blood samples); and percentage of (C) CD41^+^CD42^+^ and (D) CD41^+^CD42^−^ cells assessed by flow cytometry at Days 7, 10, 11 and 12 (*n* = 5 from *n* = 10 cord blood samples). (E) Representative dot plots of CD41^+^ and CD42^+^ Mks staining at Day 12 of culture. *p < 0.05, **p < 0.01. (F, G) After sorting CD41^+^CD42^−^ MKs at Day 7, cells were exposed to DMSO or to 4 μM YODA1 from Day 7 to Day 12 and were evaluated for: (F) percentage CD41^+^CD42^+^ Mks at D12 (*n* = 4 from *n* = 6 cord blood samples), and (G) cell proliferation (*n* = 5 from *n* = 10 cord blood samples). *p < 0.05, **p < 0.01.

We then assessed the consequences of PIEZO1 chemical activation during Mk differentiation from cord blood CD34^+^ cells. Cells were either exposed to DMSO or to increasing concentrations of YODA1 (2, 4 and 10 μM) from Day 0 to Day 12. A 1.96‐fold decrease in proliferation was observed in the presence of 10 μM YODA1 at Day 12 (*p* = 0.04), together with a significant enhanced mortality (1.44‐fold, *p* = 0.005) (Figure 2A,B). Although we observed a trend towards a lower cell proliferation with 4 μM YODA1 at D10 and D12, no significant cell proliferation reduction or excess mortality was observed at lower concentrations (2 and 4 μM) (Figure 2A,B). The absence of effect on proliferation and mortality was confirmed using MTT assay (Figure S2). For further experiments, we thus used 4 μM YODA1 concentration. Exposure of Mks to 4 μM YODA1 tended to negatively impact the megakaryocytic commitment as shown by a slight reduction in the proportion of CD41^+^ cells to 0.88 fold (*n* = 5, *p* = 0.110) at D12. Focusing more precisely on the MK maturation steps, we observed a decrease in Mk maturation, as illustrated by the percentage of mature CD41^+^CD42^+^ cells at Day 10 and 11, which decreased to 1.62 (*p* = 0.002) and 1.47 (*p* = 0.040) fold, respectively (Figure 2C,E). In contrast, the relative proportion of CD41^+^CD42^−^ immature cells increased to 1.27 (*p* = 0.090) and 1.44 (*p* = 0.029) fold, respectively (Figure 2D,E). Because the extracellular domain of GPIbα can be cleaved under culture conditions,^17^ altering the CD42b staining, these results were further confirmed using an anti‐CD42a antibody (Figure S3A,B). Finally, in order to study more specifically PIEZO1 effects on Mk maturation and to get rid of its potential early effect on early steps on haematopoiesis, we differentiated CD34^+^ cells towards Mks, sorted the CD41^+^CD42^−^ Mks at Day 7, and exposed them to 4 μM YODA1 from Day 7 to Day 12. By monitoring CD42 acquisition, we confirmed the significant decrease in the % of CD42^+^ cells at Day 12 (1.20 fold, *p* = 0.017) (Figure 2F) without significant negative impact on cell proliferation (Figure 2G).

### 3.3 PIEZO1 enhances polyploidization in megakaryocytes and PMA‐induced HEL

**FIGURE 3:**
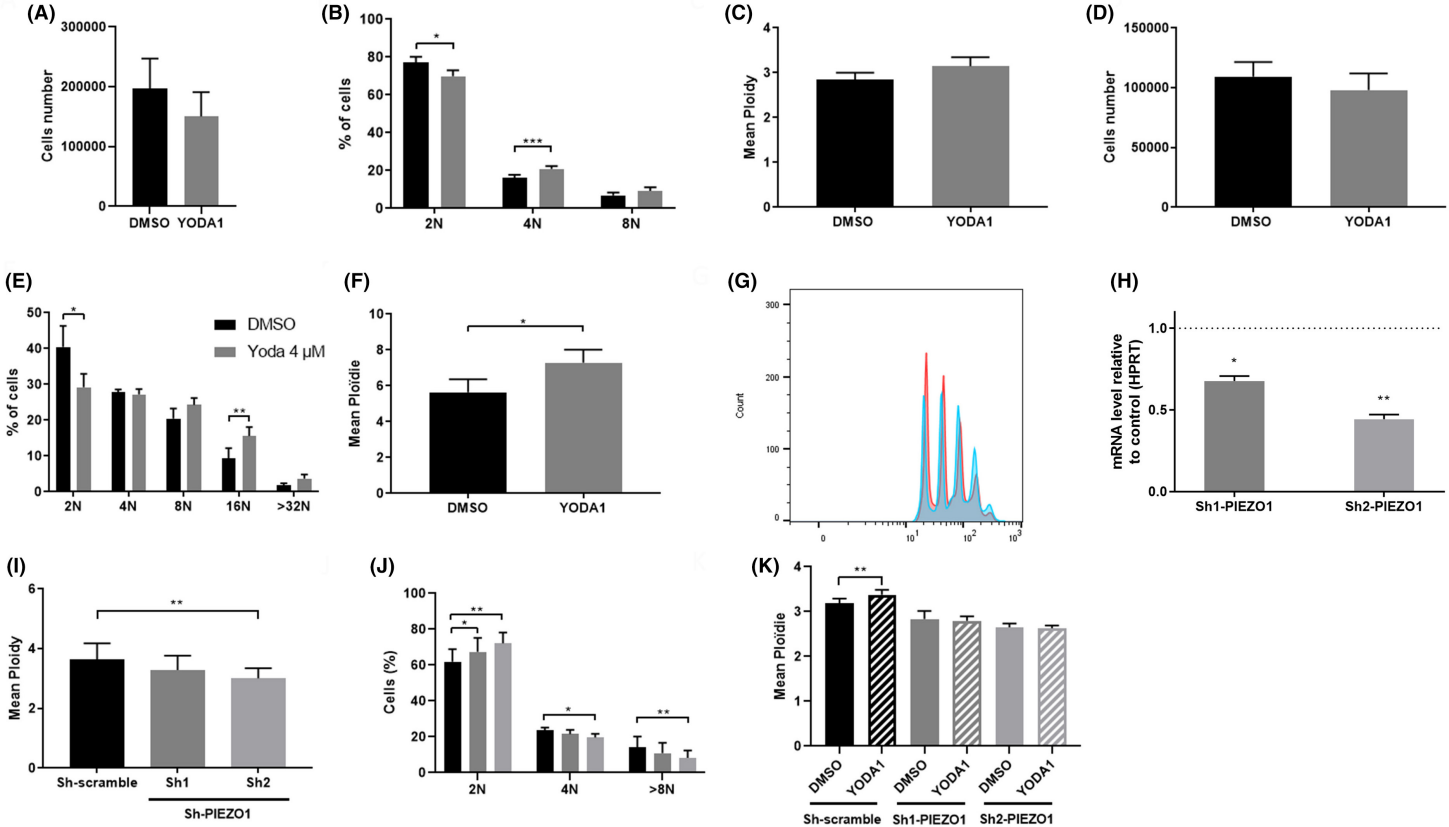
Effect of PIEZO1 activation (A–G) and silencing (H–K) on CD41^+^CD42^+^ megakaryocytes obtained from CD34^+^ originating from human cord blood (A–C) or from leukapheresis (D–K). CD41^+^CD42^+^ Mks were sorted at Day 7 and then exposed from Day 7 to 12 to DMSO (black bars) or to 4 μM YODA1 (grey bars). At Day 12 were evaluated respectively for human cord blood or leukapheresis CD34^+^ cells: (A) and (D) Cell proliferation; (B) and (E) Ploidy and (C) and (F) Mean ploidy (*n* = 3 from n = 5 cord blood or leukapheresis samples). (G) Representative histogram of the CD41^+^CD42^+^ Mks ploidy from leukapheresis culture at D12 with DMSO (in red) and upon 4 μM YODA1 activation (in blue). At Day 7, CD41^+^CD42^+^ Mks were sorted and infected with Sh‐PIEZO1 or Sh‐scramble. (H) Relative expression of PIEZO1 mRNA normalized on HPRT in primary cells transfected with Sh1‐PIEZO1 or Sh2‐PIEZO1 (*n* = 3 from *n* = 3 leukapheresis CD34^+^ samples). (I) Mean ploidy and (J) Ploidy, at Day 12 of CD41^+^CD42^+^ cells (*n* = 5 from *n* = 6 leukapheresis CD34+ samples). Similar experiments were reproduced in Mk transfected cells, and (K) Mean ploidy was evaluated (*n* = 3 from *n* = 4 leukapheresis CD34+ samples) in the presence of 4 μM YODA1 or vehicle (DMSO). *p < 0.05, **p < 0.01, ***p < 0.001.

To study the effect of PIEZO1 activation on Mk polyploidization, we sorted CD41^+^CD42^+^ Mks derived from CD34^+^ cord blood cells and exposed to 4 μM YODA1 from Day 7 to Day 12. Mortality and proliferation were not significantly impacted by YODA1 (Figure S4A, Figure 3A). PIEZO1 activation led to an increased percentage of polyploid cells, as highlighted by the increased proportion of 4 N Mks (20.8% at 4 μM YODA 1 vs. 16.2% with DMSO, *p* = 0.007) in parallel with a slight decreased percentage of 2 N Mks (69.9% at 4 μM YODA1 vs. 77.0%, *p* = 0.41) (Figure 3B). However, the mean ploidy remained low and not significantly different with or without PIEZO1 activation (Figure 3C). Considering the low capacity of cord blood‐derived Mks to reach a high ploidy in culture, we then performed the same experiments using CD34^+^ cells from adult leukapheresis. Again, 4 μM YODA1 exposure did not impact cell proliferation and viability (Figure 3D, Figure S4B). On the contrary, it increased the mean ploidy from 5.6 N to 7.3 N (*p* = 0.01) with a significant reduction of 2 N Mks (29.2% vs. 40.5%) and an increase of 16 N Mks (15.6% vs. 9.4%) (Figure 3E–G). Mean ploidy also tended to increase in a dose‐dependent manner (Figure S5A) with a maximum effect at 4 μM (with an equivalent effect at 10 μM, data not shown). To confirming the role of PIEZO1 in regulating endomitosis in human Mks, we used a shRNA‐mediated knockdown strategy. We selected two PIEZO1‐specific shRNA targeting different sequences that allowed a decrease in PIEZO1 expression by 32% and 56% at mRNA level (Figure 3H). About 25% of cells were transduced (Figure S6A). CD41^+^CD42^+^ Mks derived from CD34^+^ leukapheresis cells were sorted at Day 7, transduced with the viral supernatant and GFP^+^ cells were sorted at Day 9. At Day 11, PIEZO1 KD using the sh2 plasmid‐induced decreased the mean ploidy in comparison with the control vector (sh‐scramble) (3.0 N vs. 3.6 N, respectively, *p* = 0.006) with a significant reduction of 4 N Mks (19.6% vs. 23.8%, respectively, *p* = 0.013), 8 N Mks (8.3% vs. 14.5%, respectively, *p* = 0.014) and an increase of 2 N Mks (72.2% vs. 62%, *p* = 0.011) (Figure 3I,J). Same trends were observed with sh1‐PIEZO1 transduced cells, without reaching significance except for 2 N Mks (67.4% vs. 61.9%, *p* = 0.032), probably due to a lower efficiency in PIEZO1 KD (Figure 3J). We then assessed the mean ploidy of Mks with or without PIEZO1 KD after exposure to 4 μM YODA1 or DMSO. Mean ploidy remained increased with YODA1 (3.4 N with YODA1 vs. 3.2 N with DMSO, *p* = 0.003) in sh‐scramble transduced cells but, as expected, the effect of YODA1 on ploidy was totally abolished in cells after PIEZO1 silencing (Figure 3K).

**FIGURE 4:**
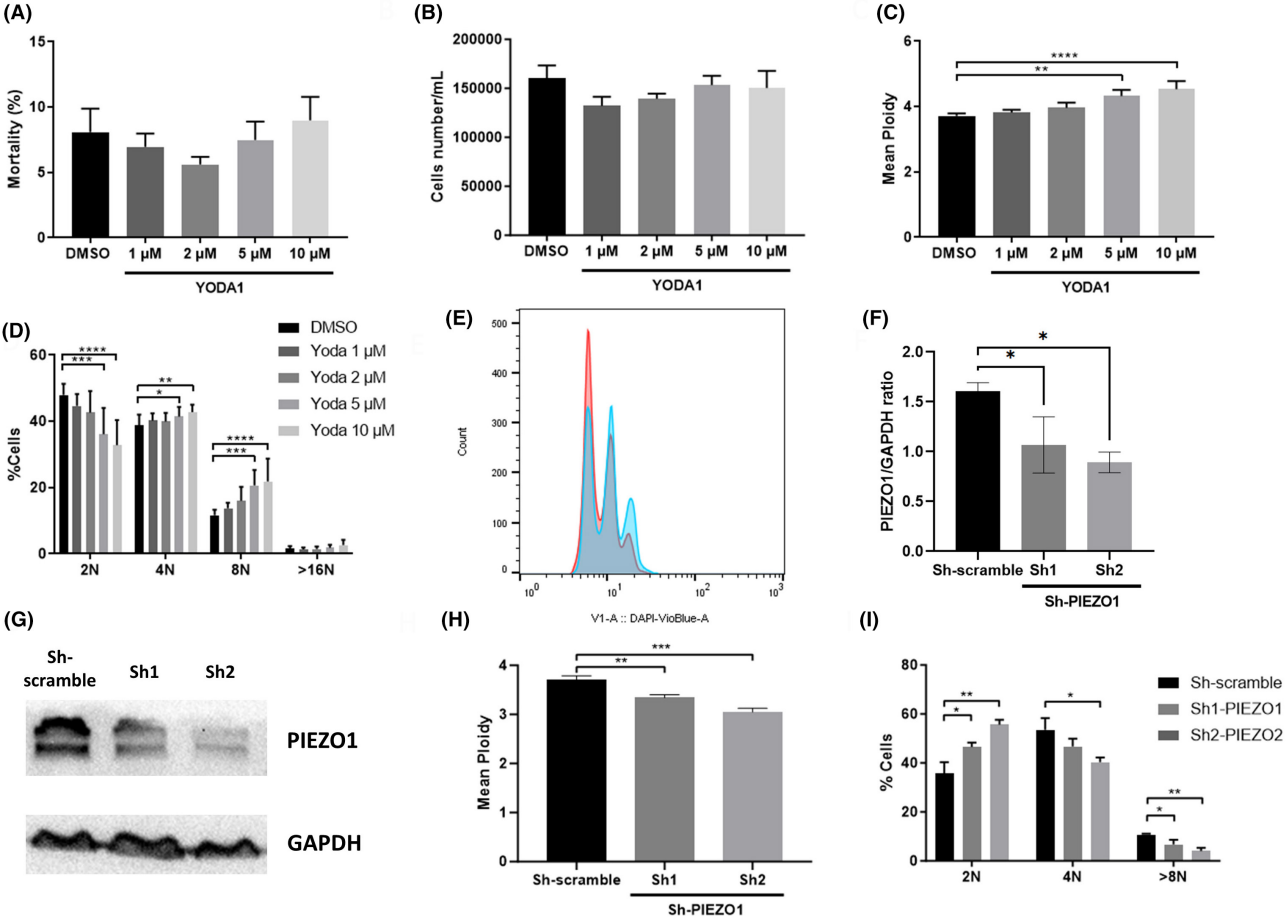
Effect of PIEZO1 activation on PMA‐induced HEL. HEL cell line was cultured with 10 nM of PMA and YODA1 concentrations ranging from 0 μM (DMSO) to 10 μM. After 48 h were evaluated: (A) Mortality rate by DAPI staining (*n* = 5); (B) Cell proliferation as assessed by cell counting in 1 mL; (C) Mean ploidy and (D) Ploïdy as cell rate. (E) Representative histogram of the PMA‐induced HEL ploidy at H48 in the presence of DMSO (control) or 10 μM YODA1. HEL cell line transducted either with Sh1‐PIEZO1, Sh2‐PIEZO1 or Sh‐control were cultivated for 48 h in the presence of 10 nM PMA and 5 μM YODA1. Then, (F and G) PIEZO1 knockdown efficiency at protein level assessed by Western blot in HEL cells (*n* = 3) as well as (H) Mean ploidy, *n* = 3 and (I) Ploidy (*n* = 3). *p value <0.05, **p value <0.01, ***p value <0.001, ****p value <0.0001.

Thus, the effect of YODA1 on ploidy requires PIEZO1 expression, confirming that this phenotype was not related to any off target effect of YODA1. Our data on ploidy were confirmed in PMA‐induced human erythroleukemia cell line HEL which differentiates into polyploid Mk‐like cells together with CD41 up‐regulation and CD235a down‐regulation^18^ in response to PMA. We exposed HEL cells to 10 nM PMA in the presence of increasing concentrations of YODA1 or DMSO as a control for 48 h. PMA did not modify PIEZO1 expression at mRNA or protein level priming (Figure S7A–C). In contrast with primary Mks, PIEZO1 activation had no significant impact on cell proliferation and survival (Figure 4A,B). However, we confirmed PIEZO1 involvement in the endomitosis process. Indeed, as observed in primary cells, PIEZO1 activation enhanced ploidy in a dose‐dependent manner (Figure 4C–E). Mean ploidy significantly increased from 3.7 N to 4.3 N (*p* = 0.002) and 4.5 N (*p* < 0.0001), after exposure to 5 and 10 μM YODA1, respectively (Figure 4C). More specifically, the percentage of 2 N cells significantly decreased from 48% to 36% (*p* = 0.0003) and to 33% (*p* < 0.0001), 4 N ploidy increased from 39% to 41% (*p* = 0.041) and 43% (p = 0.002) and 8 N ploidy increased from 12% to 21% (*p* = 0.0003) and 22% (*p* < 0.0001), at 5 and 10 μM of YODA1, respectively (Figure 4D). In PMA‐induced HEL, shRNA‐based PIEZO1 silencing decreased in PIEZO1 expression by 33% and 45% at protein level (Figure 4F,G). About 90% of cells were transduced (Figure S6B). Mean ploidy decreased significantly in comparison with cells transduced with the control vector (3.3 N, 3.0 N (*p* = 0.009) vs. 3.7 N (*p* = 0.001), respectively) (Figure 4H). More specifically, we observed a significant increase of 2 N PMA‐induced HEL (46.5% and 55.6% vs. 35.8%, respectively, *p* = 0.027 and *p* = 0.003) with a reduction of 4 N for sh2 (40.4% vs. 53.5%, *p* = 0.019) and of 8 N (6.8% and 4% vs. 10.7%, *p* = 0.037 and *p* = 0.006 for sh1 and sh2 vs. sh control, respectively) (Figure 4I).

### 3.4 PIEZO1 activation enhances proplatelet‐bearing megakaryocytes derived from CD34 ^+^ cells from human cord blood and leukapheresis

**FIGURE 5:**
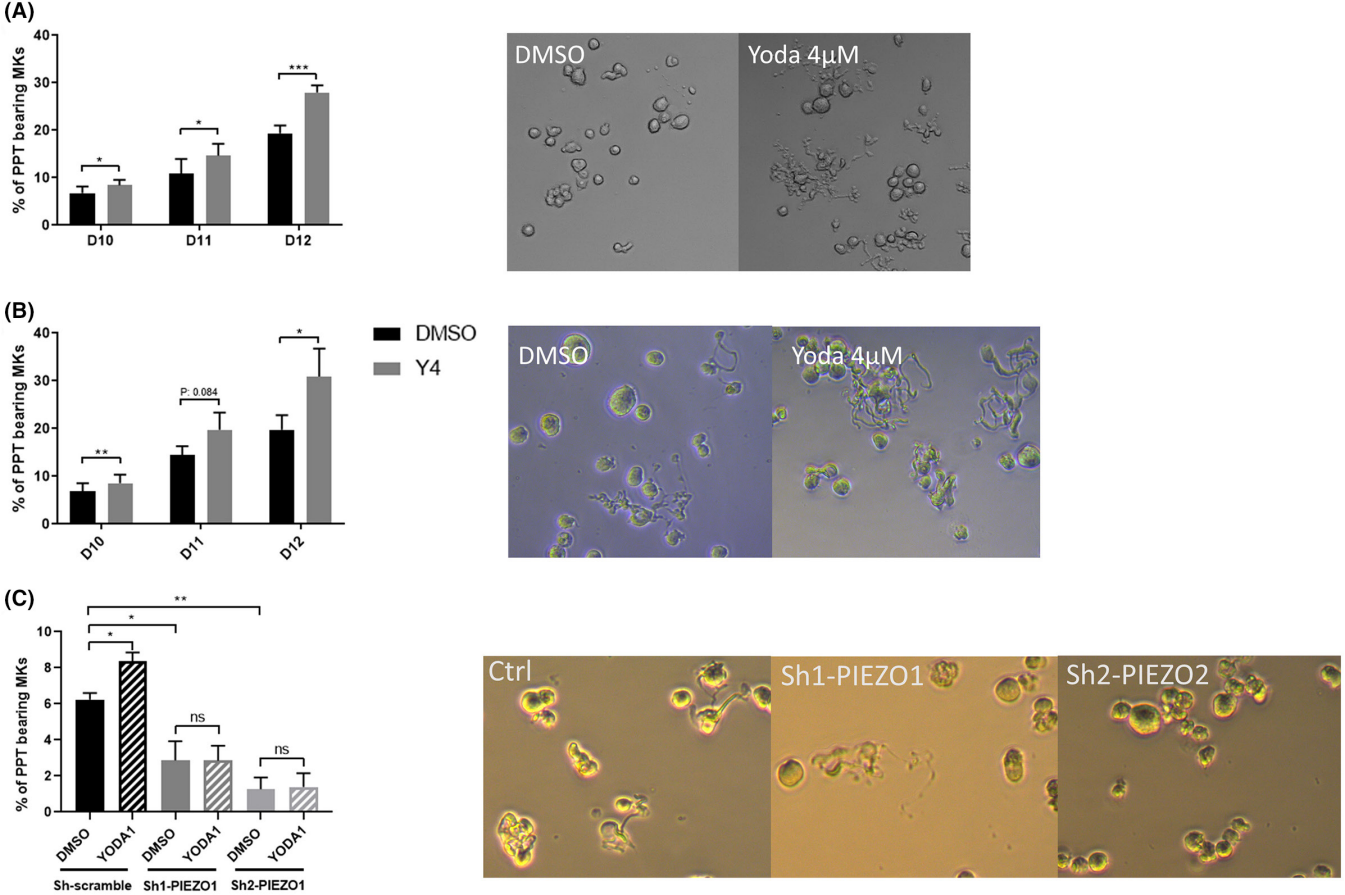
PIEZO1 activation induces proplatelet (PPT)‐bearing MKs in CD41^+^CD42^+^ Mks. At Day 7 culture of CD34^+^ cell from human cord blood (A) or leukapheresis (B and C) CD41^+^CD42^+^ Mks were sorted and plated in 96 well‐plates in the presence of vehicle (DMSO, black bars) or 4 μM YODA1 (grey bars) and, from Day 10 to 12, PPT‐bearing Mks obtained from (A) cord blood samples (*n* = 3 from *n* = 5 cord blood samples) and (B) leukapheresis samples (*n* = 4 from *n* = 4 leukapheresis samples) were quantified (Left panels). Right panels: Representative white light microscopy images of Mks with proplatelets at D12 in the presence of DMSO (control) or 4 μM YODA1. Similar experiments were performed on CD41^+^CD42^+^ Mks transducted with Sh1‐PIEZO1, Sh2‐PIEZO1 or Sh‐scramble: (C) Left panel: Percentage of PPT‐bearing MKs at D12. Right panel: Representative white light microscopy images of megakaryocytes transducted with Sh1‐PIEZO1, Sh2‐PIEZO1 or Sh‐scramble. (*n* = 3 from *n* = 3 leukapheresis samples). *p < 0.05, **p < 0.01, ***p < 0.001.

We then assessed the YODA1 effects on the number of proplatelet‐bearing Mks. CD41^+^CD42^+^ Mks obtained from human cord blood sorted at Day 7 were treated with 4 μM YODA1. YODA1 treatment increased the percentage of proplatelet‐bearing Mks at Day 10 (8.4% vs. 6.7% for control, *p* = 0.029), Day 11 (14.7% vs. 10.99%, *p* = 0.025) and Day 12 (27.9% vs. 19.4%, *p* = 0.001) (Figure 5A). Same results were found using CD41^+^CD42^+^ derived from leukapheresis: proportion of proplatelet‐bearing Mks increased at Day 10 (8.5% vs. 6.9%, *p* = 0.002) and Day 12 (30.9% vs. 19.6%, *p* = 0.029) (Figure 5B). Although not significant, the increase in platelet formation also tended to be higher at Day 11 (14.5% vs. 19.7%, *p* = 0.084). As for mean ploidy, proplatelet‐bearing Mks tended to increase in a dose‐dependent manner (Figure S5B) with a maximum effect at 4 μM (with an equivalent effect at 10 μM, data not shown). PIEZO1‐KD significantly decreased the percentage of proplatelet‐bearing Mks (2.9% (sh1), 1.3% (sh2) versus 6.2% (shSCR), *p* = 0.013 and 0.003, respectively). As expected, YODA1 exposure had no effect after PIEZO1‐KD (Figure 5C).

## 4 DISCUSSION

Mks are haematopoietic cells located in the bone marrow and the lungs, able to generate large number of platelets.^19^ They are subjected to many mechanical stimuli during their lifetime. Their size strongly increases during polyploidization, inducing membrane pressures against the surrounding cells or the extracellular matrix. Once mature, Mks extend long cytoplasmic extensions called proplatelets into sinusoid blood vessels of bone marrow. The blood flow applies pressure to the proplatelets, stretching them to generate platelets. The Mks are also in a capacity to move out the bone marrow to reach the lungs and produce platelets.^19^ In conditions of thrombocytopenia, Mks can return to the bone marrow to repopulate it in mice.^19^ These back‐and‐forth movements lead to deformations of the membranes to escape from the tissues and to enter them.

This close relationship between Mks, blood flow and the extracellular matrix makes these cells potentially particularly sensitive to mechanotransduction. In that context, we focused on PIEZO1, since it is expressed during haematopoiesis and regulates other lineages such as erythropoiesis. We show here that PIEZO1 is expressed at RNA and protein level in Mk, in agreement with published data on blood platelets and Mks.^3^, ^7^, ^9^ In Mks as in other cells, PIEZO1 activation using YODA1 increased the cytosolic Ca^2+^ level.^16^

Assessing PIEZO1 chemical activation consequences all along Mkpoiesis, we identified a specific role at the three major steps of Mk life course: acquisition of Mk markers, endomitosis and proplatelet formation. First, as previously demonstrated by Abbonante et al., it impacted negatively Mk commitment (i.e. decreased CD41^+^ expression) and early stages of differentiation (i.e. decreased switch from CD41^+^/CD42^−^ to more mature CD41^+^/CD42^+^ Mks). PIEZO1 appears to be an early regulator of haematopoiesis since a similar negative effects on differentiation were observed in erythropoiesis.^5^ Considering the highest PIEZO1 expression in CD34^+^ immature cells, before decreasing during both erythroid and Mk differentiation, one hypothesis would be that PIEZO1 maintains haematopoietic progenitors at an immature, multipotent stage, delaying induction of terminal differentiation in mature cells.

In contrast with this negative effect on early step of haematopoiesis, PIEZO1 plays a positive role in the later stages of Mk maturation. Although HEL cells lack p53 (whose absence is known to increase ploidy in megakaryocytes)^20^ and their ploidy mechanisms are poorly documented, we observed that YODA1 increased the mean ploidy level after induction of polyploidization by PMA. We then used primary mature CD41^+^CD42^+^ Mks. We observed a positive effect of PIEZO1 activation on ploidy and proplatelet formation. This is in contrast with a recent report from Abbonante et al. showing a negative effect of PIEZO1 on Mkpoiesis. However, this discrepancy may be explained by the fact that we specifically studied late Mks by sorting committed CD41^+^CD42^+^ cells, which allowed to study specifically ploidy and proplatelet formation by erasing PIEZO1 negative effects on early steps. Indeed, the important inhibition of Mk differentiation induced by PIEZO1 activation at early stages may ultimately mask on unsorted Mks, PIEZO1 positive effects on proplatelet formation and ploidy. Of note, different roles of PIEZO1 at different steps of differentiation were also observed in the erythroid lineages, where PIEZO1 regulated differentiation independently to Gardos channel in erythroid precursors whereas it regulated cell hydration through a Ca^2+^ dependent activation of Gardos in mature erythrocytes.^5^, ^21^

Our observation reinforces previous reports highlighting the role of Ca^2+^ influx and mechanotransduction in late Mkpoiesis. Indeed, on Mks, the TRPV4 mechanotransductor was shown to induce an intracellular Ca^2+^ influx in response to extracellular matrix sensing. Its chemical activation with GSK1016790A induced Akt phosphorylation and increased β1 integrin activation whereas its chemical inhibition with RN‐1734 reversed the phenotype and reduced proplatelet formation.^14^ Of interest, PIEZO1 activation (i) is associated with increased β1 integrin expression on erythroblasts^22^ and in endothelial cells^23^ (ii) plays a role upstream of TRPV4^23^ and may have the same effects on Mks.

In summary, we show here the dual role of PIEZO1 activation during Mkpoiesis, with, as previously described by Abbonante et al, an inhibitory effect during the early stages of Mk differentiation but in the present study, we also demonstrated a positive effect during late Mk maturation leading to higher ploidy and proplatelet formation. Thus, PIEZO1 could be one of Mks sensors of the environment stiffness, which is known to regulate the Mk maturation. In particular, PIEZO1 could be involved in the Mk response to a high stiffness, associated with higher ploidy, demarcation membrane development and proplatelet formation.^11^ As already observed in endothelial or osteoblastic cells and considering the similarities between PIEZO1 and TRPV4 in the positive platelet biogenesis process, one hypothesis would be that PIEZO1 acts upstream of TRPV4 to drive its opening. Thus, such as in other cell types,^23^, ^24^ PIEZO1 could induce a transient wave of Ca^2+^ flux in mature Mks which would be secondarily sustained over time by a secondary activation of TRPV4, leading *in fine* to proplatelet formation.

## AUTHOR CONTRIBUTIONS

**Julien Demagny:** Conceptualization (equal); formal analysis (equal); methodology (equal); validation (equal); writing – original draft (equal). **Sonia Poirault‐Chassac:** Conceptualization (equal); formal analysis (supporting); resources (equal). **Damtz Nehemie Ilsaint:** Resources (equal). **Aurore Marchelli:** Resources (equal). **Cathy Gomila:** Resources (equal). **Hakim Ouled‐Haddou:** Resources (equal). **Louison Collet:** Resources (equal). **Maïlys Le Guyader:** Resources (equal). **Pascale Gaussem:** Methodology (equal); resources (equal); supervision (equal); writing – original draft (equal). **Loïc Garçon:** Funding acquisition (equal); methodology (equal); project administration (equal); resources (equal); supervision (equal); validation (equal); writing – original draft (equal). **Christilla Bachelot‐Loza:** Funding acquisition (equal); methodology (equal); project administration (equal); resources (equal); supervision (equal); validation (equal); writing – original draft (equal).

## 5 FUNDING INFORMATION

This study was granted by the Agence Nationale de la Recherche (AAPG 2023 EPIOX).

## CONFLICT OF INTEREST STATEMENT

The authors declare no conflicts of interest.

## Supporting information


Figures S1–S7.

Appendix S1.

## Data Availability

Request for data should be addressed to the corresponding author.
