# Supplementary material for: Role of the mechanotransductor PIEZO1 in megakaryocyte differentiation
Source: J Cell Mol Med. 2024 Sep 20;28(18):e70055. doi: 10.1111/jcmm.70055 (PMC11415291; doi:10.1111/jcmm.70055)

**Supplemental Figure S1: Reversion of YODA1- induced calcium signal by EGTA.** Calcium influx recordings (left) and time-course of cytosolic Ca^2+^ (right) in response to 10 µM YODA1 at Day 3 of megakaryocytic culture differentiation. On the left, the histograms show fluorescence intensity over time, measured by the Ca^2+^ probe Fluo3-AM, in the presence of Ca^2+^ or in the absence of Ca^2+^ 5 mM EGTA). On the right, box plots illustrate the mean fluorescence intensity (MFI) recorded for 1 min (T=0) before the addition of YODA1 (T=0) and then again for 3 min before the final addition of the ionophore.


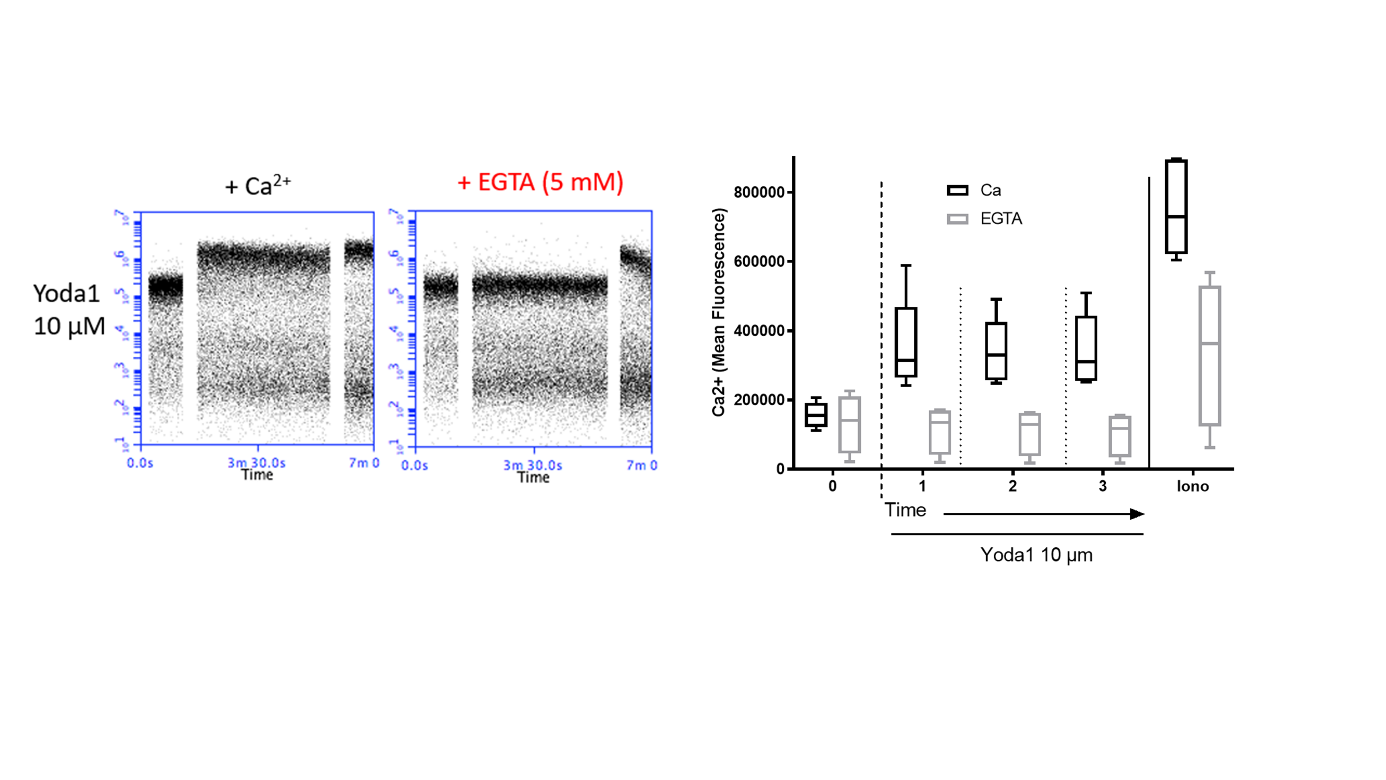


**Supplemental Figure S2: Effect of PIEZO1 activation on cell proliferation and death.** Cell cultures were exposed from Day 0 to Day 12 to DMSO (control) or to 2 µM, 4 µM or 10 µM of YODA1, and were evaluated with MTT assay at Day 12 (n=5). *: p < 0.05.


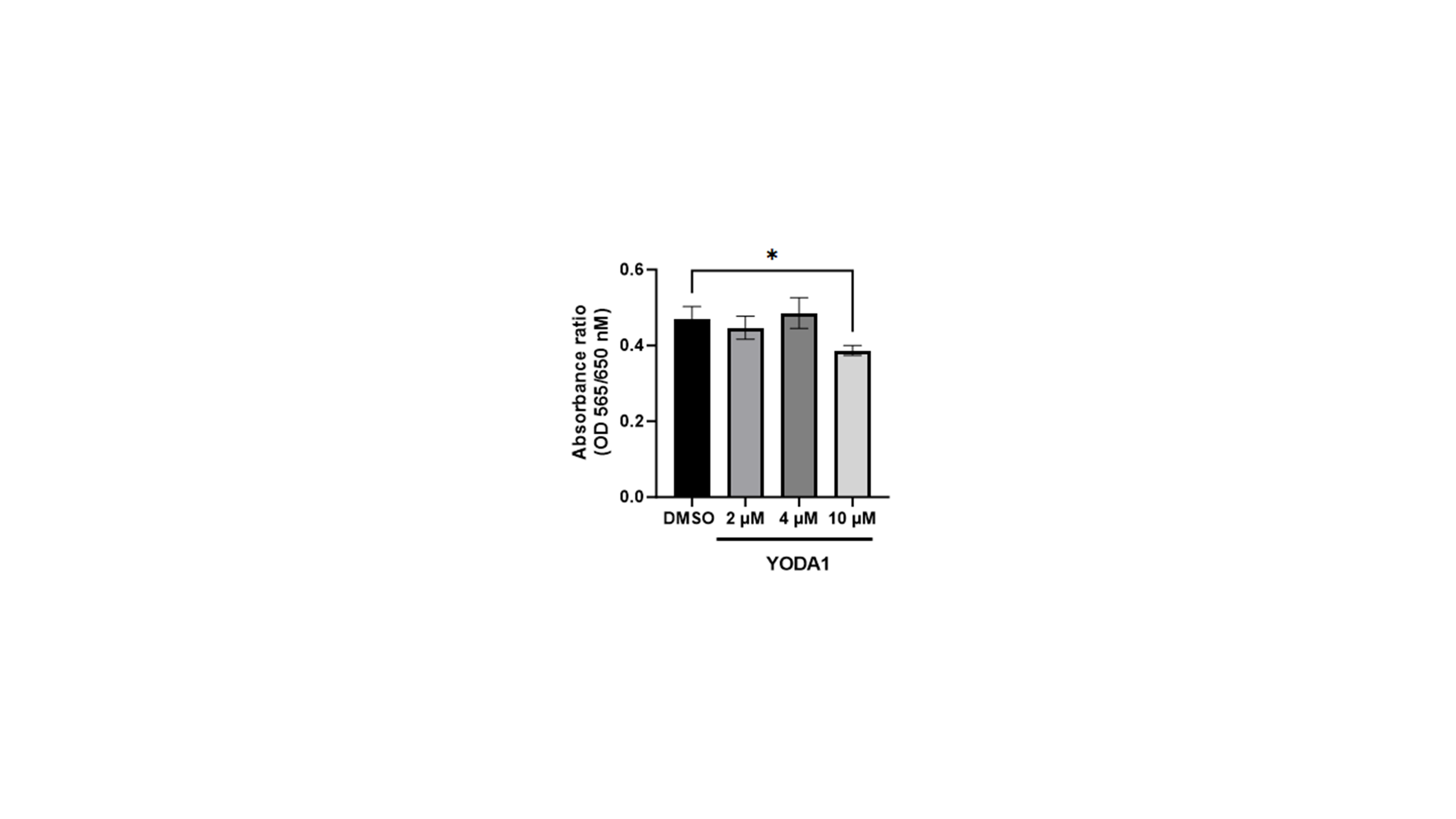


**Supplemental Figure S3: Evaluation of CD41^+^CD42^-^ and CD41^+^CD42^+^ with anti-CD42a antibody.** Cell cultures were exposed from Day 0 to Day 11 to DMSO (control) or to 4 µM of YODA1 and A) % of CD41^+^CD42a^-^ and of B) CD41^+^CD42a^+^ were evaluated by flow cytometry (n=5 from 6 cord blood samples).


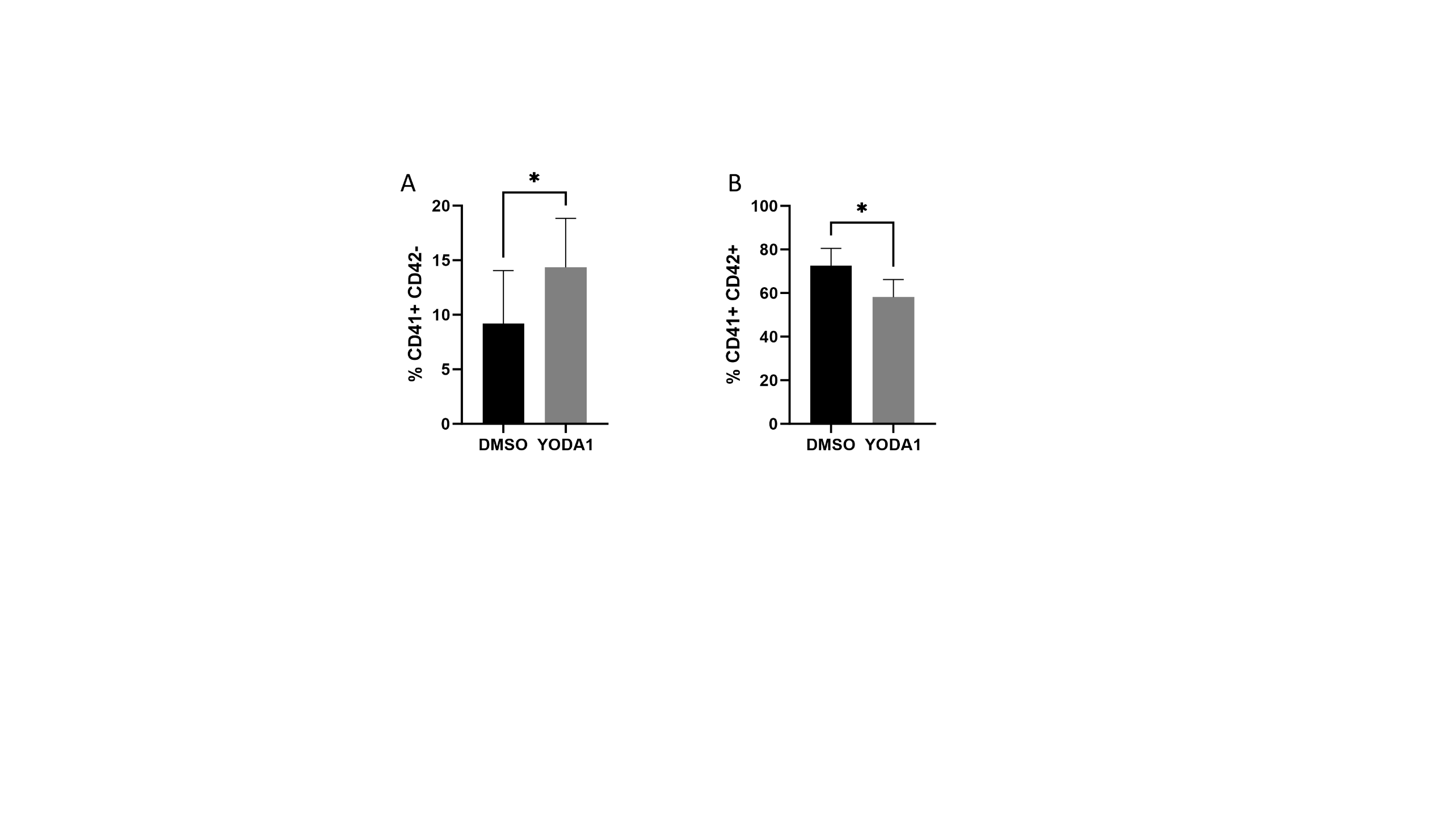


**Supplemental Figure S4: Effect of PIEZO1 activation on CD41^+^CD42^+^ megakaryocytes obtained from CD34^+^ originating from human cord blood.** CD41^+^CD42^+^ Mks were sorted at Day 7 and then exposed from Day 7 to 12 to DMSO (black bars) or to 4 µM YODA1 (grey bars). At Day 12, mortality rates, in cultures obtained from human cord blood (A) or leukapheresis (B) were assessed by DAPI staining.


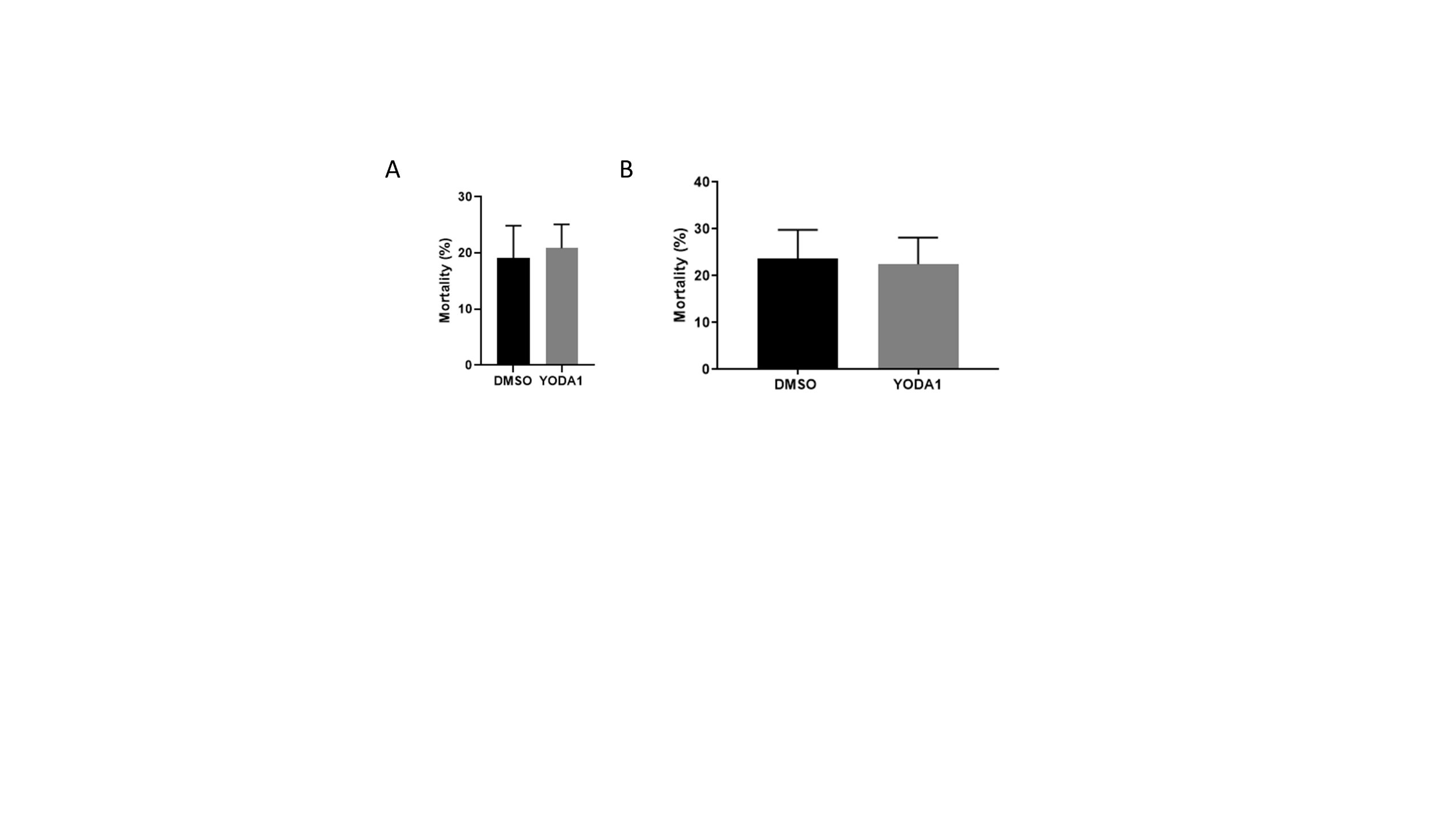


**Supplemental Figure S5: Effect of PIEZO1 activation on CD41^+^CD42^+^ megakaryocytes obtained from CD34^+^ originating from leukapheresis.** CD41^+^CD42^+^ Mks were sorted at Day 7 and then exposed from Day 7 to 12 to DMSO (black bars), or to 2 µM YODA1 (dark grey bars) or to 4 µM YODA1 (light grey bars). At Day 12 were evaluated respectively: A) Ploidy and B) proplatelet (PPT)-bearing Mks (n=4 from n=4 leukapheresis samples). *: p < 0.05, ***: p < 0.001.


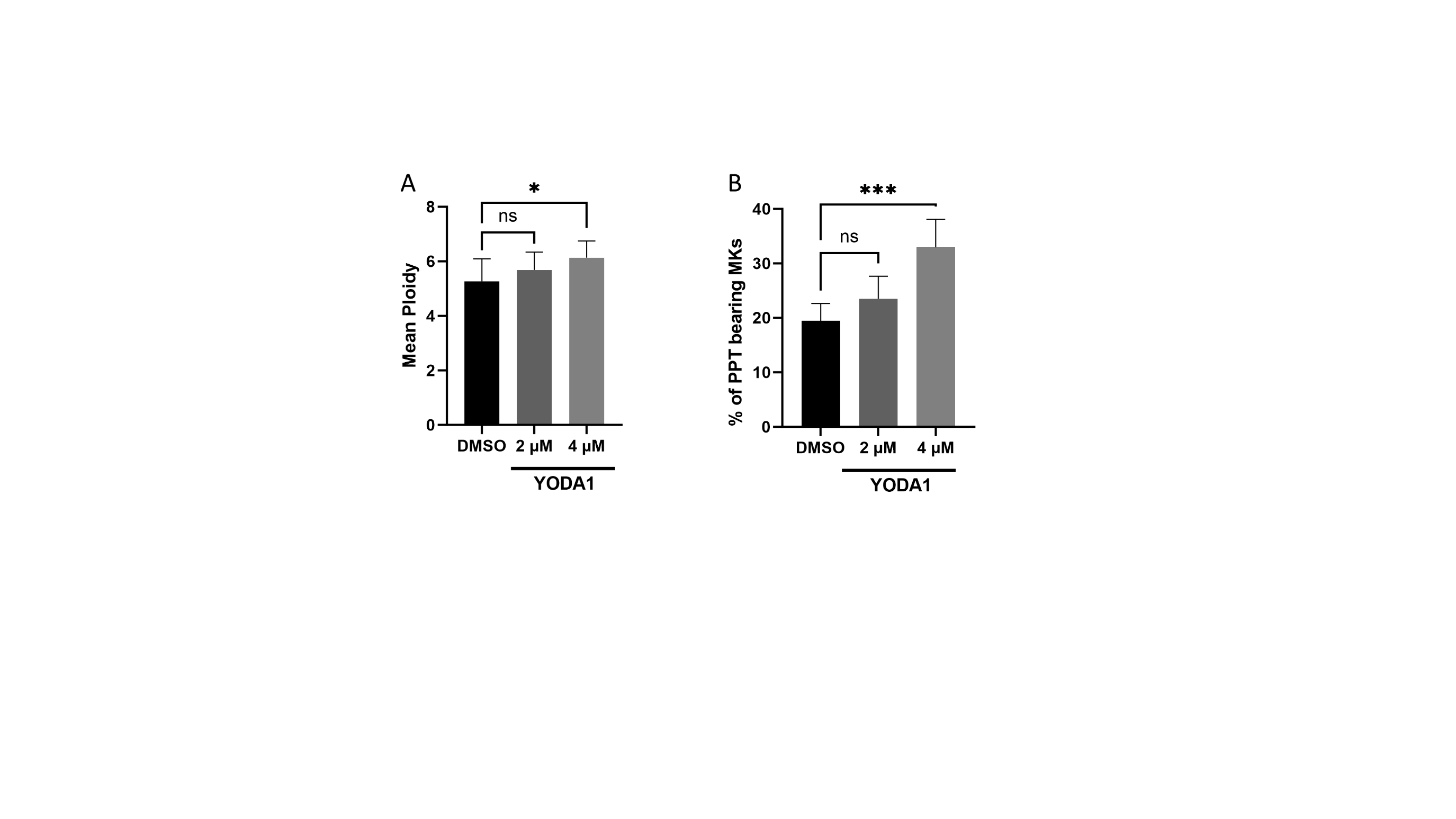


**Supplemental Figure S6: ShRNA transduction efficiency.** Percentage of GFP-positive cells after transduction of A) primary cells or B) HEL cell line with shRNA (n=4).


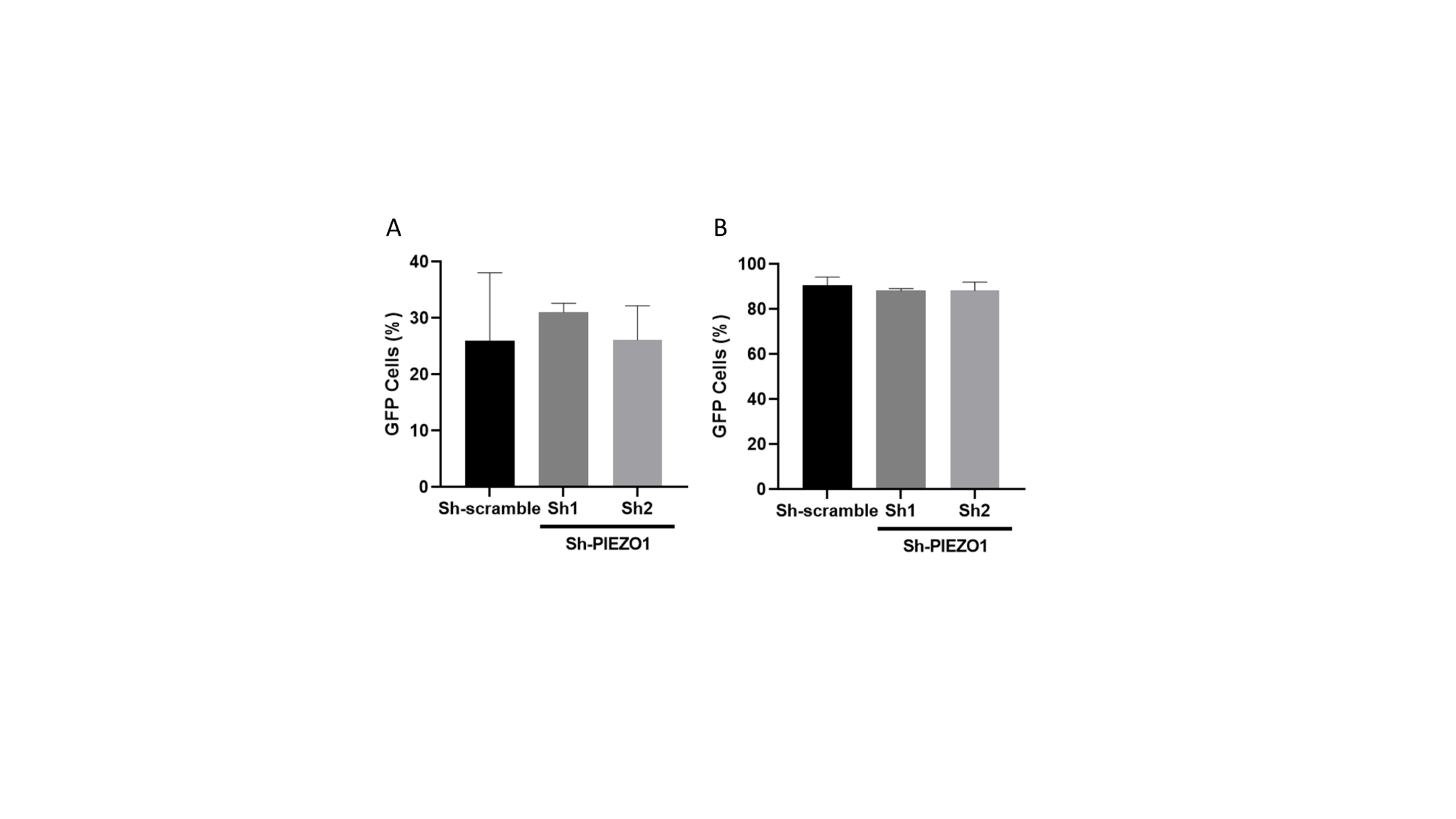


**Supplemental Figure S7: Characterization of YODA1 expression in the HEL cell line with and without PMA priming.** The HEL cells were cultured for A) 24 hours or B & C) 48 hours, followed by A) mRNA analysis and B & C) protein quantification of PIEZO1 by western-blot (n=3).


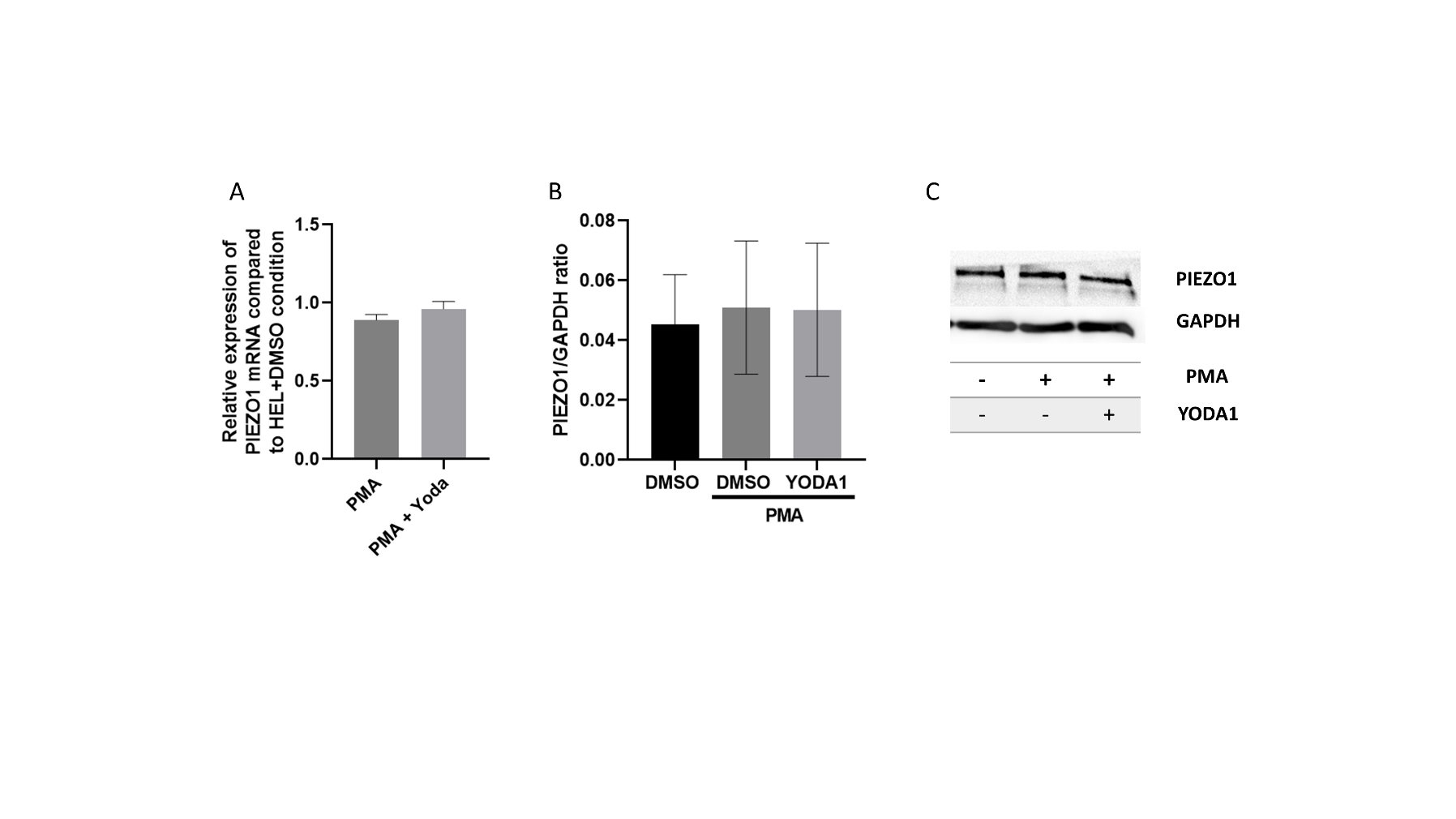

Supplement: Supplementary file 1 — Figures S1–S7. [file JCMM-28-e70055-s002.docx]
