# Supplementary material for: Role of the mechanotransductor PIEZO1 in megakaryocyte differentiation
Source: J Cell Mol Med. 2024 Sep 20;28(18):e70055. doi: 10.1111/jcmm.70055 (PMC11415291; doi:10.1111/jcmm.70055)
Supplement: Supplementary file 2 — Appendix S1. [file JCMM-28-e70055-s001.docx]

**Supplemental methods**

**Flow-Cytometry analysis and cell sorting**

Human CD34^+^ cell-derived Mks were stained with fluorescein isothiocyanate (FITC)-conjugated mouse anti-CD41a (IM0649U, Beckman coulter), phycoerythrin (PE)-conjugated mouse anti-CD42b (IM1471U, Beckman coulter), PE-conjugated mouse anti-CD42a (558819, BD Biosciences) and IgG1 (mouse)-FITC (0A07795, Beckman coulter), IgG1 (mouse)-PE (A07796, Beckman coulter) were used to assess non-specific staining. Acquisition was performed on an Accuri C6 cytometer (BD Biosciences) or a MacsQuant 8-colours flow cytometer (Miltenyi). Data analysis was performed using Accuri C6 software (BD Biosciences) and FlowJo (FlowJo v10, TreeStar Inc.). CD41^+^CD42^-^ or CD41^+^CD42^+^, depending of the experiments, were sorted on FacsAriaII device (BD Biosciences) at D7.

**Calcium ion flux assessment**

Fluo3-AM (1mM stock solution in DMSO) was added at 1:200 dilution to a cell suspension of 5.10^5^ cells/mL. Cells were incubated for 20 minutes at 37°C. The baseline was acquired for 1 minute and either YODA1, DMSO (control), EGTA, PMA, or ionophore A23187 (Sigma-Aldrich) (positive control) were added at 1/100 dilution. The Ca^2+^ flux was monitored for 4 minutes on the FL1 channel of the Accuri C6 cytometer.

**PIEZO1 western-blot analysis**

Pellet cells were extracted with RIPA buffer (R0278, Sigma) at 4°C during 30 minutes, then proteins were heated 5 minutes at 99°C with Laemmli 4X (1610747, Bio-Rad) and 10% 2-mercaptoethanol. Proteins were separated on a 10% Polyacrylamide gel in Tris-Glycine buffer (Thermo Fisher scientific) and transferred to Nitrocellulose membranes. Membranes were blocked for 1h with non-fat dry milk TBS-Tween 0.1% buffer. Membranes were incubated with the 1:1000 monoclonal anti-PIEZO1 antibody (AMAB91589, Sigma-Aldrich) overnight at 4°C in non-fat dry milk TBS-Tween 0.1%. 1:10 000 Horseradish peroxidase-conjugated secondary antibody was incubated for 1h at room temperature. Blots were visualized with chemoluminescent reagents (Super Signal Atto, ThermoFisher Scientific) in ChemiDoc Universal HoodII device (Bio-rad).

**Lenti/retroviral production and cell infection**

Two ShRNA against PIEZO1 and one control (Sh-scramble) cloned in pLKO.1-CMV-tGFP vector were purchased from Sigma-Aldrich, designed using Mission® shRNA tool. Sh1-PIEZO1 targets exon#39 (targeted sequence: GAAGACCACATCAGGTGGAA) and Sh2-PIEZO1 targets exon#51 (targeted sequence: GCACTCCATTATGTTCGAGGA). Viral production as performed in HEK293T cell line and lentiviral supernatant ultracentrifugated was titrated in HEL cells line to calculate Multiplicity of infection (MOI). HEL cells were infected overnight and harvested 48h after transduction (washed in 50ml 1X PBS) for experiments.

**Quantitative reverse transcriptase–polymerase chain reaction (RT-qPCR)**

RNA was extracted using RNeasy Plus micro and mini kit (Qiagen). Gene expression was quantified by RT-qPCR using SYBR® green on QuantStudio7 device (Applied Biosystem). HPRT was used as housekeeping gene. PIEZO1 and HPRT primers used are described in Caulier et al^5^.

**Statistical analysis**

Parametric and paired tests were used for statistical analyses and performed using GraphPad Prism software version 7.00 for Windows (GraphPad, San Diego, CA, USA). One-way analysis of variance followed by recommended post hoc multiple comparison analyses and paired two-tailed Student’s t tests were performed as appropriate. Results are shown as the mean with standard error of the mean (SEM). Asterisks indicate statistically significant differences: *p-value < 0.05, **p-value < 0.01, and ***p-value < 0.001.
